# Alteration of Trophoblast Syncytialization by *Plasmodium falciparum*-Infected Erythrocytes

**DOI:** 10.3390/microorganisms12081640

**Published:** 2024-08-10

**Authors:** Carolina López-Guzmán, Ana María García, Ana María Vásquez

**Affiliations:** 1 Grupo Malaria, Facultad de Medicina, Universidad de Antioquia, Calle 62 #52-59, Torre 1, Laboratorio 610, Medellin 050001, Colombia; 2 Escuela de Microbiología, Universidad de Antioquia, Calle 67 #53-108, Bloque 5, Oficina 5-135, Medellin 050001, Colombia

**Keywords:** trophoblast, cytotrophoblast, syncytiotrophoblast, *Plasmodium falciparum*, malaria

## Abstract

Malaria during pregnancy has been associated with significant risks to both the mother and the fetus, leading to complications such as anemia, low birth weight, and increased infant mortality. The trophoblast cells, a key component of the placenta, are crucial for nutrient and oxygen exchange between mother and fetus. The differentiation of cytotrophoblasts (CTBs) into syncytiotrophoblasts (STBs) is critical for proper pregnancy development. These cells form the bi-stratified epithelium surrounding the placental villi. While previous studies have described an inflammatory activation of STB cells exposed to *Plasmodium falciparum*-infected erythrocytes (*P. falciparum*-IE) or components such as hemozoin (HZ), little is known about the direct effect this parasite may have on the epithelial turnover and function of trophoblast cells. This study aims to contribute to understanding mechanisms leading to placental damage during placental malaria using a BeWo cell line as a differentiation model. It was found that *P. falciparum*-IE interferes with the fusion of BeWo cells, affecting the differentiation process of trophoblast. A reduction in syncytialization could be associated with the adverse effects of infection in fetal health, altering the remodeling of the trophoblast epithelial barrier and reducing their capacity to exchange substances. However, further studies are necessary to assess alterations in the functionality of this epithelium.

## 1. Introduction

Malaria during pregnancy is associated with adverse effects on both the mother and the developing fetus. Pregnant women may experience complications such as anemia, hypoglycemia, and severe malaria with manifestations like pulmonary edema, coagulation disorders, and acute renal failure [1]. Severe malaria is more common in pregnant women who live in areas with low malaria transmission and have little to no immunity against the disease [2]. Simultaneously, systemic deterioration in the mother and placental involvement during infection can lead to fetal complications, including intrauterine growth restriction [3], low birth weight [4], premature birth [5], and miscarriages [6].

A unique aspect of malaria caused by *P. falciparum* during pregnancy is the accumulation of parasites in the placenta, leading to unfavorable pregnancy outcomes. Infected erythrocytes bind to placental tissue due to the interaction between the parasitic protein erythrocyte membrane protein 1 (PfEMP-1) and the chondroitin sulfate A (CSA) expressed by syncytiotrophoblast (STB) cells [7]. Previous studies on the interaction between *P. falciparum*-IE and STBs indicated an inflammatory activation, but the role of this activation in adverse effects remains unclear, along with specific mechanisms leading to alterations in the STB barrier.

The placenta is an essential organ for proper fetal development. It is responsible for exchanging nutrients and waste products between mother and fetus, producing hormones and other molecules that regulate and maintain the pregnancy, and serving as a physical barrier separating fetal and maternal blood. Villous trophoblast cells play an essential role in these functions. In humans, the placenta is composed of villous trophoblast and extravillous trophoblast. The villous trophoblast represents the epithelial covering of the placental villous trees in direct contact with maternal blood. In contrast, extravillous trophoblasts serve additional roles, including the implantation, invasion, and remodeling of uterine arteries, to supply maternal blood to the growing fetus [8]. Villous trophoblasts have two cell populations: undifferentiated cytotrophoblasts (CTBs) and differentiated STBs [9].

STBs are a continuous, multinucleated, and specialized layer of epithelial cells that cover the entire surface of villous trees and direct contact with maternal blood. They arise from the fusion of germinative cells called CTBs. Due to its high degree of differentiation, the STB has lost its proliferative capacity [10]. The STB layer directly interacts with maternal blood, mediating nutrient, and gas exchange. It secretes several hormones, such as human chorionic gonadotrophin (hCG) and placental lactogen (PL), into the maternal circulation. These hormones are required to support the pregnancy and promote the immunological and metabolic adaptations necessary for a successful pregnancy. For instance, hCG is crucial in the early stages of pregnancy to maintain the production of progesterone and estrogens, which are essential for maintaining the uterine lining and supporting the pregnancy. Also, hCG and other placental hormones help modulate the mother’s immune response, promoting an immune environment that allows the fetus to grow. PL influences maternal metabolism, promoting the availability of nutrients for the fetus. The STBs also serve as an initial protective layer for the fetus from infection and other stressors [8,11].

Villous CTB is a precursor and proliferative cell population that supports the development of STB. These cells have high metabolic activity and are crucial in generating energy for placental maintenance [11]. CTB differentiation and fusion into STB is known as the syncytialization of trophoblast cells, and it is essential for the proper placental development and function. Any imbalance in the syncytialization may be associated with pregnancy-related diseases such as pre-eclampsia [12].

Villous trophoblast epithelia display a continuous turnover of their layers, involving CTB proliferation and differentiation to STBs, with a final apoptotic shedding event. The functional and morphological maintenance of the STB depends on the continuous turnover of this layer. A detailed description of the turnover phases involves CTB proliferating and daughter cells leaving the cell cycle and starting to differentiate. Differentiation involves cellular fusion that leads to syncytium formation and the further differentiation of STB produces hormones, transporters, and other proteins crucial for maintaining pregnancy. Finally, syncytium enters apoptosis, and apoptotic nuclei are packed into syncytial knots, which are released from the STB and enter the maternal bloodstream [10].

The differentiation of CTBs into STBs involves the activation of the expression of genes that confer the capacity to fuse with the overlying STB and other functions associated with the syncytial barrier and transport function [13]. Besides gene expression, the differentiation process is accompanied by biochemical, molecular, and morphological changes, including syncytium formation, hormonal production, and metabolic activities [14,15]. Some syncytialization markers include the production of hCG that is increased in STB, as well the expression of the endogenous retrovirus group W envelope member 1 (ERVW-1) gene, which encodes the syncytin-1 protein and ERVFRD-1 gene, which encodes the syncytin-2 protein. Both syncytin-1 and syncytin-2 participate in trophoblast differentiation and have similar structures and fusogenic properties; however, they bind to different receptors and have different localizations. Syncitin-1 is localized in STB cells, and its expression is maintained throughout gestation. Syncitin-2 is mainly localized in CTB cells, and its expression slowly decreases throughout gestation [8,16].

The BeWo cell line is a human trophoblast line derived from choriocarcinoma. It is widely used as a surrogate for CTBs to study the syncytialization process, as they can be induced to fuse and differentiate to STB [8]. BeWo cells represent a valuable model for studying the effect of maternal conditions, pathogens, or substance exposure on placental cell proliferation and differentiation pathways [17].

The interaction between *P. falciparum*-IE and the human placenta has been extensively researched, focusing on the placental immune cell alterations in response to infection. However, the impact of *P. falciparum*-IE exposure on trophoblast function and differentiation has yet to be fully elucidated. Previous studies in CTB cell lines have elucidated that components such as heme have detrimental effects on the differentiation of BeWo cells, specifically increasing apoptosis and inhibiting cell fusion [18]. This study evaluates the direct impact of *P. falciparum*-IE on trophoblast cell differentiation using the BeWo choriocarcinoma cell line, which is commonly used as a model for studying the differentiation of villous trophoblasts [19].

## 2. Materials and Methods

### 2.1. Cell Proliferation, Differentiation, and Apoptotic Markers

Cell viability was determined through MTT reduction assay and measurement of Lactate Dehydrogenase (LDH) activity in culture supernatants. Proliferation was assessed by immunostaining with an antibody against Ki67. The production of β-hCG was quantified using ELISA in culture supernatants to assess biochemical differentiation. Cell membrane fusion was evaluated with an anti-human E-cadherin antibody by immunofluorescence, and the gene expression of molecules implicated in the differentiation and fusion was quantified using qPCR. Apoptosis was measured by the immunostaining of cytokeratin 18 fragment using the M30 monoclonal antibody.

### 2.2. Antibodies

The following primary antibodies were used for immunofluorescence staining: anti-Ki67 monoclonal antibody from mouse (clone MIB-1) from Dako Cytomation (Glostrup, Denmark) at a concentration of 4 µg/mL to examine proliferation, anti-E-cadherin monoclonal antibody from mouse (anti-human E-cadherin, HECD-1) from R&D Systems (Abingdon, UK) at a concentration of 2 μg/mL to examine cell fusion, anti-M30 monoclonal antibody from mouse against the M30 epitope of cytokeratin 18 from Boehringer Mannheim GmbH (Mannheim, Germany) at a concentration of 4 μg/mL to study apoptosis, and anti-CSA monoclonal antibody from Abcam (Cambridge, UK) (ab11570) at 1:200 dilution to confirm CSA expression on the cell surface.

### 2.3. BeWo Cell Culture

The human choriocarcinoma cell line BeWo (CCL-98) was obtained from the American Type Culture Collection (ATCC) (Manassas, VA, USA). The cell line was routinely cultured in Ham F-12K medium (Sigma Aldrich, St. Louis, MO, USA), supplemented with 10% Fetal Bovine Serum (Sigma Aldrich F0926, St. Louis, MO, USA) and 1% (*v*/*v*) penicillin/streptomycin/B-amphotericin (Sigma Aldrich, St. Louis, MO, USA), following the ATCC recommendations. The BeWo cells were kept at 37 °C with 5% CO_2_. When cultures reached 70–80% confluence, they were washed with phosphate-buffered saline (PBS) 1X sterile (0780 VWR Chemicals Avantika, Jaipur, India) and detached with 0.25% trypsin-EDTA (Gibco, Billings, MT, USA) for subculture.

### 2.4. Induction of Syncytialization

Cells were seeded in 96-well plates, an 8-well Lab-TeckTM chamber (Thermo Scientific, Waltham, MA, USA), and 6-well culture plates at densities of 20,000, 50,000, and 500,000 cells per well, respectively, and allowed to adhere and adapt overnight before treatment. Cells were exposed to 25 μM or 50 μM of Forskolin (FSK) from Santa Cruz (Dallas, TX, USA), for 48 h, to induce syncytialization, and the culture medium containing FSK was renewed daily. After 48 h, the supernatants were collected and kept at −20 °C until analysis. The cells were washed with PBS and fixed in 4% paraformaldehyde (P6148, St. Louis, MO, USA) for immunofluorescent staining. In another set of experiments, cells were detached using trypsin, and RNA was extracted and stored at −80 °C for gene expression analysis using RT-PCR.

### 2.5. Culture of P. falciparum-IE Strain Adherent to CSA (FCB1CSA)

The FCB1CSA strain was grown in A+ erythrocytes using RPMI-1640 medium (Sigma Aldrich, St. Louis, MO, USA), which was supplemented with 25 mM HEPES, 21.6 mM NaHCO_3_, 16 µg/L of gentamicin, 0.2 mM of hypoxanthine (Sigma Aldrich, St. Louis, MO, USA), and 10% A+ human serum. Continuous cultures were maintained at a hematocrit (Hto) of 5% in a gas mixture of 5% CO_2_, 5% O_2_, and 90% N_2_, at 37 °C [20]. The parasitemia was monitored daily, and the medium was changed every 24 h. Healthy erythrocytes were added thrice a week until a 5–7% parasitemia level was achieved [20,21]. To preserve the CSA adherent phenotype of parasites, panning selection on CSA was performed every four weeks, as described previously [21].

### 2.6. Concentration of Mature Forms of P. falciparum by Gelatin

The gelatin flotation was used to concentrate mature stages of *P. falciparum* (trophozoites and schizonts) from cultures. Parasite cultures were centrifuged at 2500 rpm for 5 min. The culture supernatant was removed, and 1% porcine gelatin solution (Sigma Aldrich, St. Louis, MO, USA) in incomplete RPMI-1640 was added to the *P-falciparum*-IE pellet (1 volume of IE pellet/ten volumes of gelatin). The mixture was incubated at 37 °C for 40 min, allowing separation into two phases. The upper phase, containing the mature stages, was then transferred to a new tube and washed twice with incomplete RPMI-1640 by centrifugation at 2500 rpm for 5 min. The parasitemia was estimated by counting *P. falciparum*-IE in a thin blood smear [20,21].

### 2.7. Plasmodium falciparum Cytoadherence Assay

Cytoadherence assays were conducted to measure the number of adherent IEs to BeWo cells using static conditions [21]. The cells were grown in 8-well Lab-Tek^®^ II chambers (Nunc^®^, St. Louis, MO, USA), until they reached a confluence of 50–80% over 2–5 days. Confluent cultures were treated with 50 μM FSK for 48 h to induce syncytialization. Unsyncytialized BeWo cells were used as a control (BeWo-CTBs). After treatment, cells were washed using an adhesion medium (RPMI 1640 supplemented with 0.5% BSA) at pH 6.7. Subsequently, they were incubated with 300 μL of a mature stage IEs suspension with a parasitemia of 10% and hematocrit of 1% in an adhesion medium. The incubation was carried out for one hour at room temperature with constant agitation (100 rpm). After incubation, non-adherent erythrocytes were removed by washing the slides four times with an adhesion medium. The adhered IEs were fixed overnight in 2% glutaraldehyde and then stained for 45 min with 1% Giemsa. The level of adhesion was quantified microscopically by counting the number of attached IEs to a total of 500 STB nuclei [21].

### 2.8. Exposures of BeWo Cells to Plasmodium falciparum-IE

The impact of *P. falciparum*-IE on BeWo cells was assessed by co-exposing the cells to *P. falciparum*-IE and FSK. Unsyncytialized BeWo cells were used as a control. Cells were plated and allowed to adhere to culture plates overnight. The following day, they were washed and simultaneously treated with 50 µM FSK and a parasite suspension for 24 h. Subsequently, parasites were removed, and the cells were incubated once again for an additional 24 h in the presence of 50 µM FSK to complete 48 h of FSK treatment. The parasite suspension was prepared at a parasitemia of 10% and a hematocrit of 3%, using mature stages of parasites (trophozoites and schizonts) recovered from culture through gelatin flotation [21].

The specificity of the adherence was determined by blocking the interaction using soluble CSA (sCSA) (Sigma Aldrich, St. Louis, MO, USA). To block the ability of IEs to bind to BeWo cells via CSA, the parasite suspension was incubated with 100 μg/mL of sCSA for 30 min before the adhesion assay. After the blocking, the parasite suspension was added to the BeWo cells culture in lab-tech chambers to perform the adherence assay as previously described. The inhibition percentage of adherence was determined as follows: 100 − (number of IEs adhered in the presence of soluble CSA/number of IEs adhered in control) × 100 [21].

### 2.9. Measurement of Cell Viability by MTT Reduction

This test evaluates the ability of healthy cells to reduce MTT (3-[4,5-dimethylthiazol-2-yl]-2,5-diphenyltetrazolium bromide) to formazan, as an indicator of mitochondrial activity. The MTT test was performed as described by Mosmann in 1983 [22] and used to evaluate the cytotoxic effect of FSK. Briefly, BeWo cells were seeded at a density of 20,000 cells per well in 96-well plates. Cultures were washed and the cells were treated with 25 and 50 µM FSK for 48 h. After incubation, 10 μL of 5 mg/mL MTT (Alfa Aesar, Haverhill, MA, USA) solution in PBS was added to each well and incubated at 37 °C for 4 h. Then, the culture medium was removed, and 130 μL of dimethyl sulfoxide (DMSO) (Sigma Aldrich, St. Louis, MO, USA) was added to dissolve the formazan crystals. Finally, the absorbance in each well was measured at 570 nm using a spectrophotometric microplate reader (Multiskan™ FC Microplate Photometer, Thermo Scientific™, Waltham, MA, USA).

### 2.10. Measurement of Cell Viability by LDH Activity

The LDH is a cytosolic enzyme released into the extracellular environment, indicating cell damage or lysis. The “Cytotoxicity detection kit” (Roche Diagnostics GmbH, Mannheim, Germany) was used to detect the enzymatic activity and performed in accordance to the manufacturer’s instructions. After 24 h of culture, 100 µL of the supernatant was transferred to a 96-well plate in triplicate. Then, 100 µL of the reaction mixture (catalyst and dye solution) from the kit was added to each well. The plate was incubated in darkness at room temperature for 30 min. After that, 50 µL of 2 N sulfuric acid (R&D Systems, Minneapolis, MN, USA) was added to stop the reaction. The optical density (OD) was measured at 450 nm using a microplate reader (Multiskan™ FC Microplate Photometer, Thermo Scientific™ Waltham, MA, USA). Triton X-lysed tissue was used as a positive control to validate the technique, representing 100% LDH release.

### 2.11. Immunofluorescence Staining

Immunofluorescence staining was performed following the general procedures previously published [17,18]. The proper antibody dilutions were determined before conducting the experiments, following the manufacturer’s instructions. Cells were seeded at a density of 50,000 cells per well in Lab-Tek^®^ II chambers and exposed to different treatments. After washing with 1X PBS, cells were fixed with 4% paraformaldehyde. Subsequent washing was performed with PBS, followed by simultaneous permeabilization and blocking with Triton X100 and PBS-BSA 10%. The primary antibody in PBS-BSA 1% was added, and cells were incubated at 4 °C overnight. After washing with PBS 1X, cells were incubated with the secondary antibody using the Alexa-488 fluorescence system (Molecular Probes, Eugene, OR, USA) and Hoechst (Molecular probes, Eugene, OR, USA) for nuclear counterstaining. Stained cells were preserved in Fluorsave^®^ (Sigma Aldrich, St. Louis, MO, USA) and analyzed using a Zeiss Axio Vert.A1 fluorescence Microscope (Oberkochen, Germany). Images were collected with a Zeiss AxioCam Cm1 (Oberkochen, Germany). Notably, permeabilization was necessary only for Ki67 and M30 staining. M30 staining was performed after 72 h of FSK treatment to evidence apoptosis processes.

### 2.12. Analysis of Immunofluorescence Staining

#### 2.12.1. Cellular Fusion

BeWo cells were stained with E-cadherin to highlight cell borders and with a nuclear stain to visualize the nuclei. The number of nuclei in syncytia and the total number of nuclei were counted in ten randomly selected fields. The fusion index (FI) was calculated using the formula: (total number of nuclei in syncytia/total number of nuclei) × 100 [23].

#### 2.12.2. Cell Proliferation and Cell Death

The percentage of positive cells for Ki67 and M30 was determined by counting the cells emitting fluorescence among the total number of stained nuclei in ten randomly selected fields.

### 2.13. RNA Extraction and cDNA Synthesis

BeWo cells were seeded in 6-well plates at 500,000 cells/well in 2 mL of culture medium. The cells were allowed to attach for 24 h before any treatment was added. Once treated, the medium was removed and stored, and the cells were subjected to RNA extraction using the RNeasy PLUS Mini Kit from Qiagen (Hilden, Germany), following the manufacturer’s instructions. The RNA concentration and purity were determined using a Nanodrop 2000 UV-Vis Spectrophotometer (Thermo Fisher Scientific, Waltham, MA, USA). The RNA was then stored at −80 °C until used. To synthesize cDNA, 1 μg of total RNA was used, and reverse transcription was carried out using the qScriptTM SuperMIX^®^ cDNA synthesis kit from QuantaBio (Beverly, MA, USA), as per the manufacturer’s protocols. The thermic profile of the reaction was 5 min at 22 °C, followed by 30 min at 42 °C and 5 min at 85 °C, then a cool-down to 4 °C. The cDNA was stored at −20 °C until further use.

### 2.14. Real-Time PCR

The HOT FIREPol^®^ EvaGreen^®^ qPCR Supermix (QuantaBio, Beverly, MA, USA) was used for the PCR reaction under universal amplification conditions, following the manufacturer’s instructions. The reaction was carried out on the AriaMx Real-Time PCR System (Agilent, Santa Clara, CA, USA) and included the following steps: 12 min at 95 °C, followed by 15 s at 95 °C, 30 s at 62 °C, and 30 s at 72 °C. The final reaction volume was 20 μL, consisting of 2 μL of cDNA, 12.5 μL of Master mix (Solis Biodyne, Tartu, Estonia), and 1.125 μL of primers, adjusted with 5.25 μL of water. The delta–delta CT method was used to determine the relative expression, with GAPDH as the normalization gene. The primer sequences are shown in Table 1.

### 2.15. Detection of hCG in Culture Supernatants

Following the manufacturer’s recommendations, the hCG hormone, mainly produced by the STB, was measured using the DuoSet ELISA enzymatic immunoassay kit (R&D Systems, Minneapolis, MN, USA) to detect free protein in the culture supernatant. The absorbances were measured in a Microplate Photometer (Multiskan™ FC Thermo Scientific™, Waltham, MA, USA) at 450 nm OD. The concentration of hCG was determined in pg/mL by extrapolating the OD data from a standard curve.

### 2.16. Statistical Analyses

The experimental data were presented as the mean ± standard error of the mean (SEM). A repeated ANOVA test was employed to compare groups, and Tukey’s post hoc test was applied to compare conditions. Statistical significance was set at * *p* < 0.05, ** *p* < 0.01, and *** *p* < 0.001. Statistical analyses were performed using GraphPad Prism version 10.

## 3. Results

### 3.1. Evaluation of the Optimal Conditions to Induce Cell Differentiation

Optimal conditions for BeWo cell differentiation were established using FSK stimulation (25 and 50 μM). Cytotoxicity was measured by MTT reduction and LDH levels as surrogates of cytotoxicity and by hCG production as a marker of chemical differentiation and endocrine function (Supplementary Figure S1). The fusion of BeWo cells was studied using E-cadherin immunostaining to evaluate cell membrane fusion under treatment with two concentrations of FSK by 48 h. Syncytium formation was determined by the fusion of three or more nuclei and was calculated using the fusion index.

**Figure 1:**
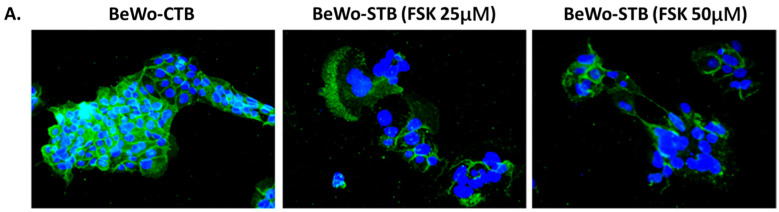

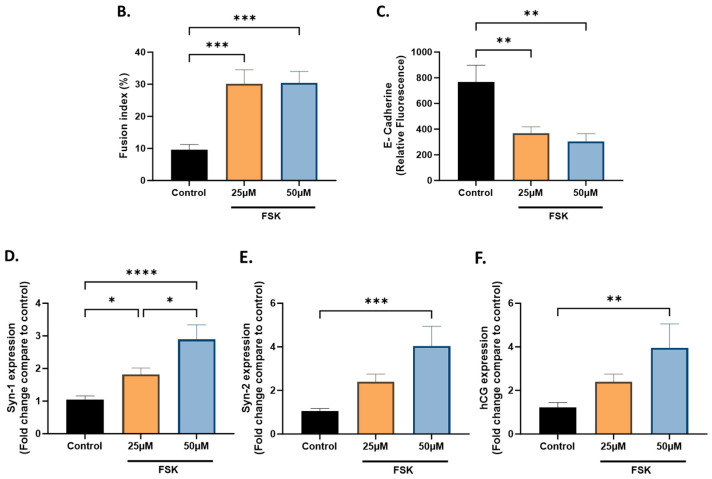
BeWo cell fusion in the presence of FSK. (**A**) Immunofluorescence staining for E-cadherin (green) and nuclei (blue) of cultured BeWo cells under treatment with FSK (25 μM and 50 μM, 400×). (**B**) Mean fluorescence intensity of E-cadherin in control cells and FSK treatments. (**C**) Syncytialization index in control cells and FSK treatments. (**D**) Expression of SYN-1 showed a 2.65-fold increase in cells treated with 50 μM FSK compared to the control. (**E**) Expression of SYN-2 showed a 4.04-fold increase in cells treated with 50 μM FSK compared to the control. (**F**) Expression of βhCG gene indicated a 3.32-fold increase in cells treated with 50 μM FSK compared to the control (n = 3). Data are presented as mean ± SEM. ANOVA, *p*-value: <0.00001 (****), <0.0001 (***), <0.001 (**), and <0.005 (*).

In untreated BeWo cells, there was a low level of spontaneous fusion, and most cells were in the mononucleated state. FSK induced the fusion of BeWo cells, forming multinucleated syncytia and reducing E-cadherin staining (Figure 1A–C). The expression of three genes (SYN-1, SYN-2, and βhCG) involved in the syncytialization and differentiation of trophoblast was evaluated by qPCR. A significant increase in the expression of these molecular markers was observed when BeWo cells were exposed to 50 μM of FSK (Figure 1D–F).

Other parameters related to epithelial turnover, such as trophoblast proliferation and apoptosis, were evaluated to establish the proper conditions for studying these processes in BeWo cells exposed to FSK (Supplementary Figure S2).

An immunofluorescence labeling of CSA was carried out to test if the 25 µM and 50 µM FSK treatment changes the expression of the CSA used by *P. falciparum* for adherence. The expression of CSA remained similar across different treatments, including the control BeWo-CTB cells (Supplementary Figure S3). A cytoadherence assay was performed to ensure that CTB and STB mediated the FCB1CSA *P. falciparum* strain’s CSA-specific adherence. Both CTB and STB support the adherence of infected erythrocytes. The reduction in cytoadherence observed after the pre-incubation of infected erythrocytes with soluble CSA indicates that BeWo cells specifically supported the binding of parasites through CSA (Supplementary Figure S4).

### 3.2. Effect of P. falciparum-IE in the Cell Viability and Proliferation Rate during Syncytiotrophoblast Differentiation

**Figure 2:**
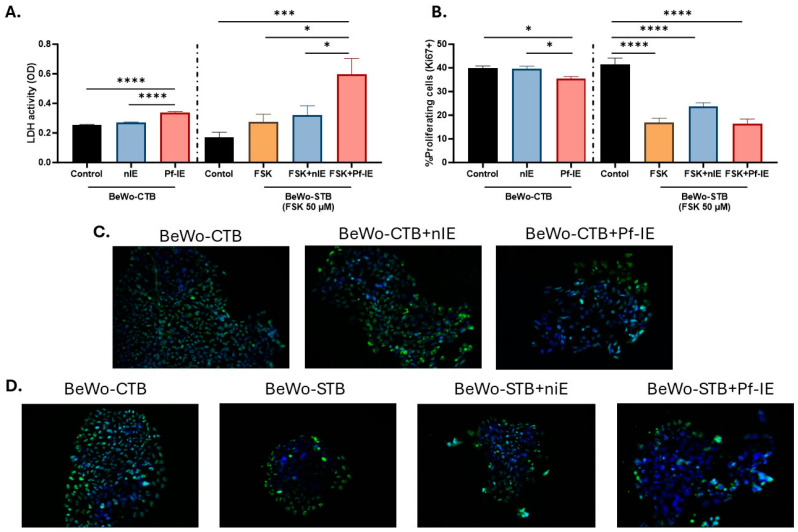
Cell viability and proliferation in BeWo cells exposed to *P. falciparum*-IE. (**A**) LDH activity was measured in the culture supernatant of BeWo-CTB and BeWo-STB cells exposed to infected erythrocytes as a marker of cytotoxicity. (**B**) Percentage of proliferative cells (Ki67-positive cells) in BeWo-CTB and BeWo-STB cultures exposed to *P. falciparum*-IE. (**C**) Representative images of Ki-67 (green) and nuclei (blue) immunostaining in BeWo-CTB (100X). (**D**) Representative images of Ki-67 (green) and nuclei (blue) immunostaining in BeWo-STB (100X). Results are presented as mean ± SEM (n = 3). Test: ANOVA, *p*-value: <0.00001 (****), <0.0001 (***) and <0.005 (*).

Cell damage induced by the exposure to *P. falciparum*-IE was studied by measuring LDH levels. Our findings revealed a significant role of the parasite in inducing cellular damage in BeWo-CTB and BeWo-STB (Figure 2A). Optical density (OD) levels that reflect LDH activity increased when BeWo-CTB cells were exposed to the infected erythrocytes, changing from 0.253 ± 0.04 in control to 0.338 ± 0.07 in treated cells. Cell viability was also compromised in BeWo-STB cells after interaction with infected erythrocytes (OD 0.59 ± 0.10), as compared to the controls: BeWo-STB alone (0.27 ± 0.05, *p*-value = 0.0148), BeWo-STB exposed to non-infected erythrocytes (OD 0.31 ± 0.06, *p*-value = 0.0423), and BeWo-CTB (0.17 ± 0.04, *p*-value = 0.0008) (Figure 2A).

The nuclear antigen Ki67 is present in all actively proliferating cells, including trophoblasts. Thus, the frequency of Ki67-positive nuclei was used as a surrogate for the proliferative state of cultures under treatment (Figure 2B). The changes in the proliferation rate in BeWo-CTB cells in response to *P. falciparum*-IE were evaluated, and it was observed that the cell exposure of *P. falciparum*-IE induced a modest decrease in the proliferation compared to control (35.5% versus 40%, *p* < 0.05) (Figure 2C). Also, the changes in the proliferation rate during syncytiotrophoblast differentiation in response to *P. falciparum*-IE were evaluated (Figure 2D). In control conditions, 41.4% of BeWo cells stained positive to Ki-67, and treatment with FSK reduced the number of proliferative cells (16, 9%, *p* < 0.0001). Furthermore, the exposure of BeWo cells to FSK and *P. falciparum*-IE does not affect the lower proliferation rate observed in the differentiated cells (BeWo-STB). The percentage of proliferating cells was 16.99 ± 1.75% and 16.39 ± 2.02% in STB and STB exposed to parasites, respectively (Figure 2B).

### 3.3. Plasmodium falciparum-IE Reduces BeWo Cells Fusion

**Figure 3:**
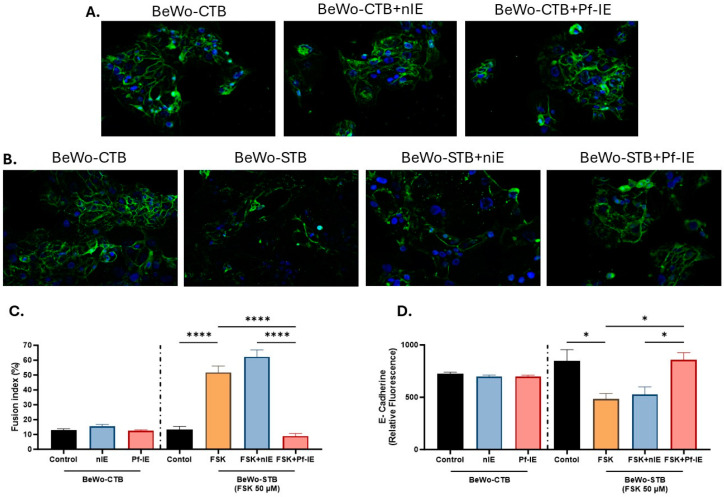
BeWo cell fusion is altered by exposure to *P. falciparum*-IE. Immunofluorescence staining for E-cadherin (green) and nuclei (blue) of BeWo-CTB cells (200X) (**A**) and BeWo-STB (200X) (**B**) cells under parasite treatment. (**C**) Fusion index under different stimulatory conditions. (**D**) Mean fluorescence intensity of E-cadherin different stimulatory conditions. Data are presented as mean ± SEM (n = 3). Test: ANOVA, *p*-value: <0.00001 (****), <0.005 (*).

The differentiation and fusion of CTB are crucial for a healthy pregnancy. Consequently, the impact of infected erythrocytes on BeWo cell fusion was assessed. Initially, a low level of spontaneous fusion was observed in BeWo-CTB cells, serving as a control, with most of the cells remaining in the mononucleated state (13% fusion index). Treatment with non-infected and infected erythrocytes did not significantly affect the spontaneous fusion of BeWo-CTB cells (15.6% and 12.5% fusion index, respectively) (Figure 3).

Forskolin stimulated the fusion of BeWo cells to form multinucleated syncytia while also reducing E-cadherin immunofluorescence staining. Notably, *P. falciparum*-IE significantly impacted the syncytialization process, inhibiting the fusion process. FSK triggers a fusion index of 51.7% and decreased E-cadherin expression in BeWo cells after 48 h of treatment (Figure 3). However, when BeWo cells were simultaneously exposed to parasitized erythrocytes and FSK, the fusion index was 9%, with no reduction in E-cadherin expression. These results indicate that *P. falciparum*-IE inhibited the Forskolin-induced cell fusion of BeWo cells.

### 3.4. Plasmodium falciparum-IE Decreases the Expression of Syncytialization Markers

It was explored whether parasite exposure induced changes in the expression of trophoblastic differentiation markers, such as as SYN-1, SYN-2, and hCG. Since there were no significant changes in BeWo-CTB cells without Forskolin (FSK) stimulation, the subsequent analyses were only conducted in BeWo cells induced to differentiate through FSK treatment.

**Figure 4:**
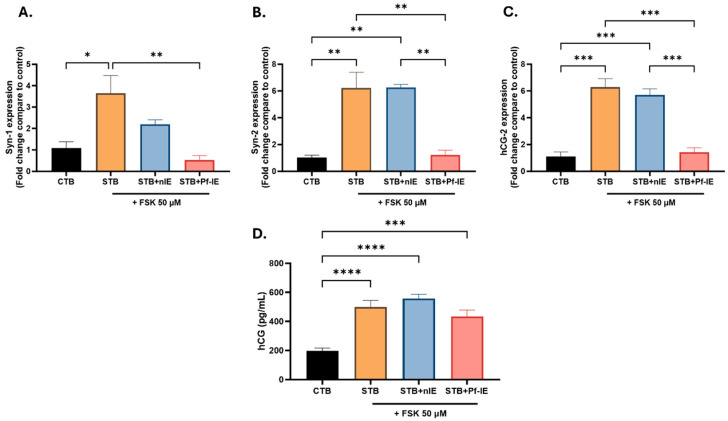
Expression levels of the SYN-1, SYN-2, and βhCG genes in BeWo-STB cells under parasite treatment, evaluated by qPCR. (**A**) The SYN-1 gene expression. (**B**) The SYN-2 gene expression. (**C**) The βhCG gene expression. (**D**) hCG levels in culture supernatants BeWo cells exposed to FSK and infected erythrocytes (n = 3). Data are presented as mean ± SEM. Test: ANOVA, *p*-value: <0.0001 (****), <0.001 (***), <0.01 (**), <0.05 (*).

A significant decrease in the expression of genes related to trophoblast differentiation, such as the SYN-1, SYN-2, and hCG, was observed when the STB was exposed to *P. falciparum*-IE, reaching expression values like those found in the CTB (Figure 4). The expression of the SYN-1 gene exhibited a three-fold decrease in STB exposed to *P. falciparum*-IE compared to STB (*p*-value 0.0063) (Figure 4A). The expression of the SYN-2 gene showed a five-fold decrease in STB exposed to *P. falciparum*-IE compared to STB (*p*-value 0.0010) (Figure 4B). The βhCG gene demonstrated an approximately five-fold decrease in expression in STB exposed to *P. falciparum*-IE compared to STB (*p*-value 0.0004) (Figure 4C). The amount of hCG protein in the culture supernatant was measured, and it was found that BeWo-STB produces higher levels of hCG than BeWo-CTB, which was expected. Treating BeWo cells with both FSK and *P. falciparum*-IE resulted in a slight decrease in hCG production, but the difference was not significant (Figure 4D). Nevertheless, this supports the findings at the gene expression level.

Based on these results, *P. falciparum*-IE disrupts the cellular fusion process in the CTB to STB differentiation pathway, as evidenced by the increased expression of E-cadherin in STB exposed to parasites and the low syncytialization index. Notably, a significant decrease in the expression of crucial molecular mediators in the fusion process, such as SYN-1, SYN-2, and hCG, was observed, further emphasizing the negative impact of *P. falciparum*-IE on the CTB differentiation process.

### 3.5. Plasmodium falciparum-IE Does Not Increase Apoptosis during Syncytiotrophoblast Differentiation

**Figure 5:**
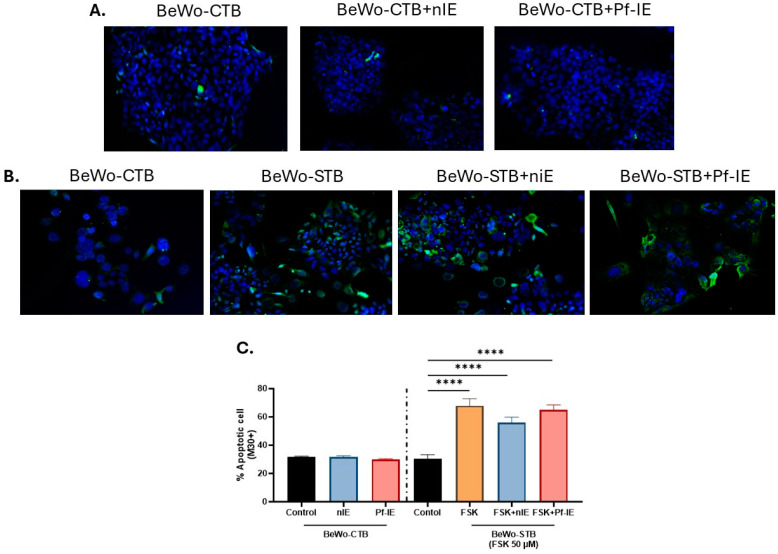
Quantification of BeWo apoptotic cells (M30-positive) exposed to *P. falciparum*-IE. (**A**) Panel of photographs of M30 staining (green) and nuclei (blue) in BeWo-CTB under different study conditions (200X). (**B**) Panel of photographs of M30 staining (green) and nuclei (blue) in BeWo-STB under different study conditions (200X). (**C**) Percentage of apoptotic cells in control cells and STB exposed or not to *P. falciparum*-IE. Data are expressed as mean ± SEM (n = 3). Test: ANOVA, *p*-value: <0.00001 (****).

The M30 antibody recognizes a neoepitope of cytokeratin 18 formed by caspase-3 cleavage and thus was used to evaluate the apoptosis in the epithelial cells. Based on the number of M30-positive cells, under control conditions, BeWo cells showed a rate of apoptosis of 30%, which increased to 60% under FSK treatment, indicating higher apoptosis in STB-differentiated cells than in undifferentiated CTB. The higher apoptotic rate of STB remains unaffected after exposure to *P. falciparum*-IE. The percentage of cells stained positive to M30 was 67.8 ± 4.9% and 64.82 ± 3.69% in STB and STB exposed to parasites, respectively (Figure 5). The treatment of BeWo-CTB cells to parasites without co-treatment with FSK did not affect the apoptosis rate.

## 4. Discussion

The mechanism behind *P. falciparum* placental malaria is primarily attributed to the ability of infected erythrocytes to adhere to placental tissue through the interaction between VAR2CSA and CSA on the surface of syncytiotrophoblast (STB), along with an inflammatory response characterized by the infiltration of monocytes and macrophages into the placenta, leading to the subsequent production of cytokines and other inflammatory mediators [24]. It is noteworthy that while this mechanism has been extensively studied over the years [25,26,27,28], limited research has been conducted on the direct impact of *P. falciparum*-IE on the STB, the target cells of cytoadherence. This research aimed to understand the effects of *P. falciparum*-IE on trophoblast cells, particularly on the process of epithelial turnover (CTB-STB), which is vital for facilitating maternal–fetal gas and nutrient exchange.

Interestingly, it was found that *P. falciparum*-IE inhibits cellular fusion, a critical step in STB differentiation, as evidenced by a decreased cellular fusion index and increased expression of E-cadherin, indicating the maintenance of intercellular junctions. Additionally, these findings were validated by the significant decrease in the expression of Syn-1 and Syn-2, which are crucial regulators in mediating fusion pore opening, completing membrane fusion, and cellular content mixing [29,30]. Consistent with these findings, previous studies have described that the decreased expression of these genes negatively regulates the cell cycle detection process that CTB cells undergo and the subsequent fusion process [31,32], primarily affecting Syn 1 and 2 [33,34].

The experimental design presented in this study does not allow us to conclude how *P. falciparum*-IE might alter cell fusion. It can be hypothesized that a molecule expressed on the surface of the infected erythrocyte or released after schizonts rupture, such as GPI, HZ, or even the heme group, plays a significant role as an inhibitor or disruptor in this process and is associated with increased cellular apoptosis presence [18]. We propose that these molecules are candidates that negatively influence the activation of the protein kinase K-dependent pathway, which has been shown to affect cell fusion without compromising hCG production [8,35]. Regarding the endocrine function, a notable decrease in hCG expression and a trend toward lower levels in the supernatant of cells cultured with *P. falciparum*-IE were observed. This suggests a potential impact on STB function due to inadequate differentiation.

The interaction of villous trophoblast with other pathogens such as *Cytomegalovirus* (CMV) [36,37,38], *Toxoplasma gondii* [39,40], and *Trypanosoma cruzi* [41,42,43] have been extensively studied. It has been reported that these pathogens have a direct negative impact on STB integrity and function and modulate cytokine, chemokine, and proapoptotic factors production. Regarding epithelial turnover, previous studies have shown that human CMV and Zika virus (ZIKV) infections can suppress trophoblast syncytialization [37,44].

CMV suppresses the syncytialization of primary cytotrophoblasts and interferes with the gene expression profile related to trophoblast differentiation [37]. ZIKV infection can disrupt the structure of mature trophoblast organoids and inhibit syncytialization. Since the trophoblast plays fundamental functions in the placenta, it might be a factor associated with placental dysfunction [44]. Complementing these findings, CMV-infected placenta from pregnancies with fetal growth restriction showed CTB accumulation and arrested differentiation, suggesting that the disruption of syncytialization by CMV-infected CTBs reduces the villi’s capacity to exchange substances, leading to restricted fetal growth due to congenital CMV infection during pregnancy [38].

Contrary to described before, the protozoan parasite *T. cruzi* has been associated with increased trophoblast turnover. In vitro studies have reported that *T. cruzi* can induce trophoblast differentiation, cellular proliferation, and apoptotic cell death. These changes may increase the epithelial turnover, proposed as a local antiparasitic mechanism in the human placenta [40,41]. Consequently, trophoblast cells may be less susceptible to *T. cruzi* infections than other cells, such as fibroblasts, that do not undergo epithelial turnover [42]. In placental tissue from women with chronic Chagas disease, it has been observed a significant decrease in the CTB proliferation and fusion and an increase in the trophoblast detachment and apoptosis in placental villi, suggesting that the renovation of the trophoblast would be altered in placentas from women with chronic infection [43].

The discrepancy with the in vitro studies in *T. cruzi* highlighted that some observations could differ from those in vivo, where other factors contribute to the pathological consequences. Together, the data suggest an increase in the epithelial turnover at the beginning of the interaction between the parasite and trophoblast. However, chronic exposure to parasites may reinforce the continuous turnover and eventually induce alterations in the dynamics of trophoblast renewal that might directly affect fetal development [43].

The rate of congenital transmission of *T. gondii* is higher than that of *T. cruzi*; although there is no information on the effect of this apicomplexan parasite on trophoblast differentiation. It is well described that the infection induces severe placental tissue damage, altering the placental barrier and villous stroma of the placental villi, and increases the proinflammatory response [45,46].

The mechanisms of action of these pathogens compared to *P. falciparum* may be different since their interactions with the placental epithelium vary significantly. For example, *P. falciparum* does not invade the STB; it merely adheres to it, whereas *T. cruzi* and *T. gondii* do invade it, with the latter being more invasive. Some of the most noticeable effects that differ between *T. cruzi* and *P. falciparum* are that the former promotes cellular differentiation [42,43], which has been interpreted as a potential part of a response or defense mechanism of the trophoblast epithelium to avoid parasitic invasion [41]. It is essential to mention that there may be differences in the pathophysiology between strains of the parasites, and this is an interesting question to address in the future. For example, whether *P. falciparum* strains with different levels of adherence or var2csa expression might have a different impact on cellular fusion [47].

The limitations of this study included the exclusive use of BeWo cells. This cell line is a recognized model for studying the fusion process; however, it is essential to highlight that cancer-derived cell lines are transformed, and thus, the use of primary trophoblasts has the advantage of representing normal cells. Also, BeWo cells exhibit low levels of spontaneous syncytialization, and most studies that evaluate the impact of external stimuli (pathogens, chemicals, others) stimulate cells with Forskolin, which induces elevated cAMP levels to increase cell–cell fusion. A limitation concerning cell proliferation measurements was the lack of synchronized BeWo cell cultures, which is a vital process in the study of cells progressing through the cell cycle because cell synchronization is a process that coordinates the division of cells in a culture so that they all divide at the same time [48].

The impairment of cellular fusion is a critical step that disrupts syncytial differentiation and function. If STB formation is dysregulated, syncytial regeneration is interrupted, thereby compromising the integrity of the placental exchange surface, which can be detrimental to maternal and fetal health and is associated with pregnancy complications such as intrauterine growth restriction [49]. In summary, it is essential to highlight that the in vitro model with BeWo cells identified an adverse effect of *P. falciparum*-IE on trophoblast cell fusion. Trophoblast fusion is crucial in the placenta for maintaining a healthy pregnancy, as it is essential for preserving the STB layer, the direct interface between maternal blood and fetal tissues [50]. The further exploration of the molecular mechanisms mediating this damage is vital for implementing future strategies to prevent placental function deterioration and subsequent fetal health impairment.

## 5. Conclusions

Infected erythrocytes by *P. falciparum* disrupt the cellular fusion process in the CTB to STB differentiation pathway, evidenced by the expression of E-cadherin and the low syncytialization index. Notably, a significant decrease in the expression of crucial molecular mediators in the fusion process, such as SYN-1, SYN-2, and hCG, was observed, further emphasizing the negative impact of *P. falciparum*-IE on the CTB differentiation process. Moreover, it was also found that *P. falciparum*-IE causes cellular damage, as the levels of LDH in culture supernatants were higher in the STB exposed to parasites. An alteration in the fusion of mononuclear CTB could be associated with adverse pregnancy outcomes, as has been described in other pathologies.

## Figures and Tables

**Table 1:** The primer sequences of the syncytialization mediators used for qPCR.

Gen	Forward Primer Sequence (5′-3′)	Reverse Primer Sequences (5′-3′)
ERVW-1 (Syncityn-1)	GCA ACC ACG AAC GGA CAT C	GTA TCC AAG ACT CCA CTC CAG C
ERVFRD-1 (Syncytin-2)	CGG ATA CCT TCC CTA GTG CC	AGC TGA GGT TGC TGG TTC TG
βhCG	GCT ACT GCC CCA CCA TGA CC	ATG GAC TCG AAG CGC ACA TC
GAPDH	GGT GTG AAC CAT GAG AAG	CCA CGA TAC CAA AGT TGT C

## Data Availability

The original contributions presented in the study are included in the article and Supplementary Materials, further inquiries can be directed to the corresponding author.

## Supplementary Materials

The following supporting information can be downloaded at: https://www.mdpi.com/article/10.3390/microorganisms12081640/s1. Supplementary Figure S1: Viability of BeWo cells after treatment with FSK measured by MTT assay, LDH activity, and βhCG production; Supplementary Figure S2: Decreased proliferation and incremented apoptosis in cells that differentiate into syncytiotrophoblast; Supplementary Figure S3: CSA expression in BeWo-CTB and BeWo-STB cells; Supplementary Figure S4: Syncytiotrophoblast and cytotrophoblast cells support *P. falciparum*-IE cytoadherence through chondroitin sulfate A.
