# Supplementary material for: Alteration of Trophoblast Syncytialization by Plasmodium falciparum-Infected Erythrocytes
Source: Microorganisms. 2024 Aug 10;12(8):1640. doi: 10.3390/microorganisms12081640 (PMC11356531; doi:10.3390/microorganisms12081640)
Supplement: Supplementary file 1 [file microorganisms-12-01640-s001.zip › microorganisms-3101949-supplementary.pdf]

Supplementary data

Supplementary Figure S1. Viability of BeWo cells after treatment with FSK measured by MTT assay, LDH activity, and βhCG production.

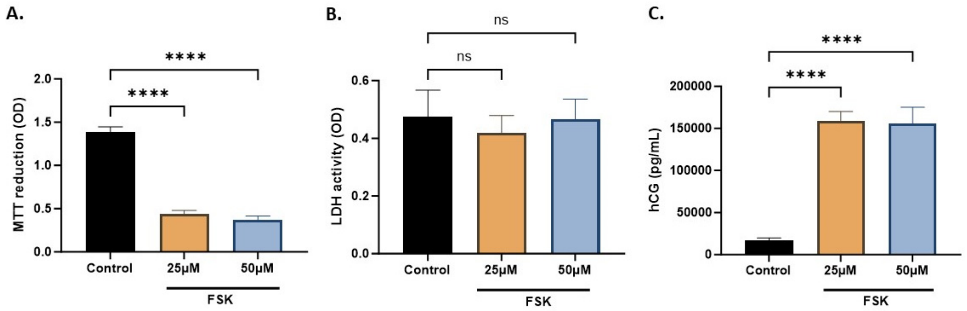

Supplementary Figure S1. Viability of BeWo cells after treatment with FSK measured by MTT assay, LDH activity and βhCG production. A. MTT reduction as an indicator of mitochondrial activity (n=3). B. Measurement of lactate dehydrogenase activity as an indicator of cell viability. OD: Optical densities of control and treated cells (n=3). C. Comparison of βhCG production in pg/mL based on the concentration of FSK stimulus used in STB vs. control CTB cells (n=3). ANOVA p-value: <0.0001 (****).

After performing the MTT assay, it was observed that nearly half of the cells showed a decrease in mitochondrial activity compared to the control cells or unstimulated (CTB) cells. This observation could have two possible explanations. Firstly, FSK may be causing cell death. Secondly, FSK might trigger cellular differentiation, decreasing cell proliferation and significantly reducing mitochondrial activity. Notably, the number of active mitochondria is directly proportional to the number of cells in the assay. Hence, a higher number of cells would result in more active mitochondria. As mentioned earlier, cellular differentiation from CTB to STB involves cell cycle arrest, and cell fusion is associated with the non-replicative differentiation of STB (S. Figure 1A). To determine if FSK impacted cell viability, we measured LDH activity. The results showed no significant differences in LDH activity in the supernatants of cells exposed to the treatment versus control cells (S. Figure 1B). Therefore, it is suggested that the observed decrease in mitochondrial activity in the MTT assay was not due to cell death or damage. Instead, it probably resulted from the cell differentiation process. Differentiated STB produced an average of 157,382 + 45,483 pg/mL of hCG compared to CTB cells, which produced an average of 17,104 + 8,336 pg/mL of this hormone. These data suggest that the STB are actively performing their function in the specific case associated with the production of the hormone hCG, a primary characteristic of well-differentiated STB, data shown in the S. Figure 1C.

Supplementary Figure S2. Decreased proliferation and incremented apoptosis in cells that differentiate into syncytiotrophoblast.

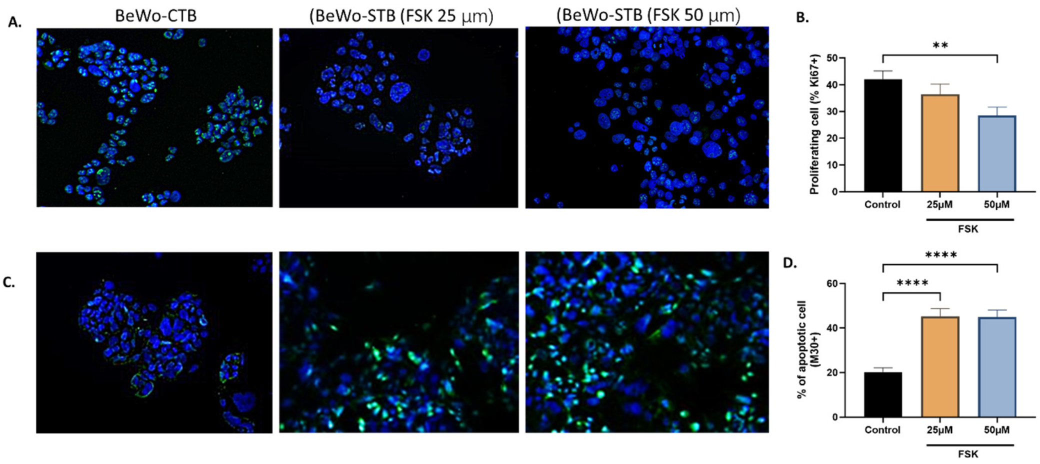

Supplementary Figure S2. The epithelial turnover leads to a decrease in proliferation and an increase in apoptosis. A. Immunofluorescence staining for Ki67 (green) and nuclei (blue) of cultured BeWo cells under treatment with FSK (25μM and 50μM, 200X). B. Mean fluorescence intensity of Ki67 in control cells and FSK treatments. C. Immunofluorescence staining for M30 (green) and nuclei (blue) of cultured BeWo cells under treatment with FSK (25μM and 50μM, 200X). D. The frequency of apoptotic cells increases in cells treated with 50 μM FSK compared to the control. (n= 3). Data are presented as mean±SEM. ANOVA, p-value: <0.001(**); <0.00001 (****).

The impact of FSK stimulation on the cellular proliferation process was assessed, and it was found that, as expected, when CTB differentiated into STB, given that the latter is non-proliferative, Ki67 expression decreased. A significant reduction in the expression of Ki67 with FSK was observed, and the presented data corresponded to three independent assays conducted on different days. (S. Figure 2A-B). In the final step of cell differentiation, non-proliferative STB undergoes cell death. To determine the frequency of cells undergoing apoptosis, M30 staining was employed. M30 staining detects a neoepitope that results from cytokeratin 18 cleavage during cell death. The study found that as STB differentiates, cell death increases, as expected in the final differentiation process. There was a significant increase in M30 expression in differentiated cells, with an average of apoptotic cells at 45% ± 2.06, compared to the control with an average of apoptotic cells at 20.26% ± 10.1 (p-value < 0.0001) (S. Figure 2C-D).

Supplementary Figure S3. CSA expression in BeWo-CTB and BeWo-STB cells.

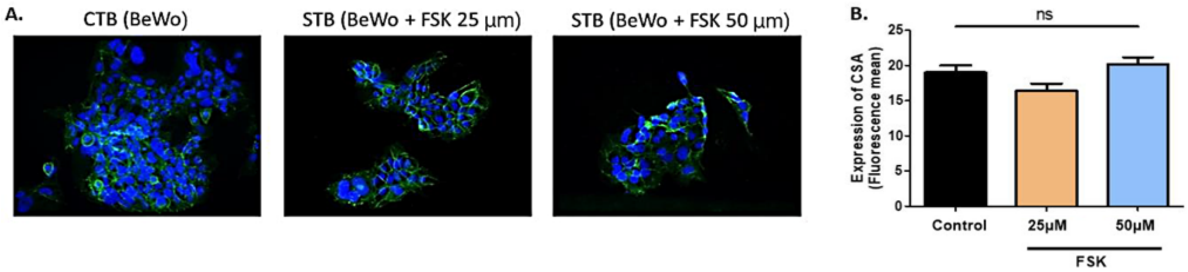

Supplementary Figure S3. Evaluation of CSA expression in BeWo cells. A. Photographic record from CSA expression in control BeWo cells and treated with 25μM and 50μM FSK. In green: staining for CSA, in blue: Hoechst for nuclei. B. Mean fluorescence intensity of CSA in control cells and FSK treatments. (n=3).

Supplementary Figure S4. Syncytiotrophoblast and cytotrophoblast cells support *P. falciparum*-IE cytoadherence through chondroitin sulfate A

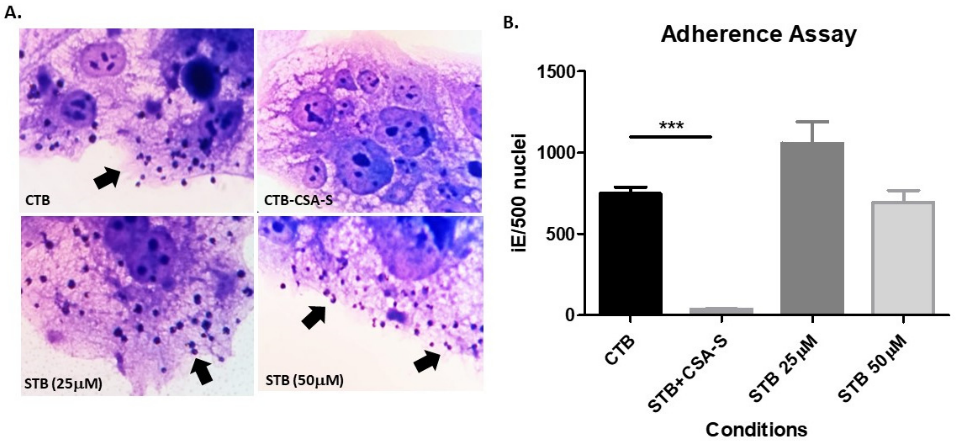

Supplementary Figure S4. The cytoadherence of *P. falciparum*-CSA+ is supported by cytotrophoblast cells and syncytiotrophoblast. A. Representative images of *P. falciparum* FCB1-CSA cytoadherence on BeWo cells treated with 25µM and 50µM forskolin (STB), untreated control cells (CTB), and specificity control cells with preincubation of soluble CSA, stained with Giemsa. Parasitized erythrocytes with *P. falciparum* adhered to the cells are indicated (black arrow). B. Statistical frequency of the cytoadherence assay, using soluble CSA as the specificity control (CTB+Soluble CSA). (Total magnification of 1000X). (n= 3). Data are presented as mean ± SEM. ANOVA p-value: < 0.05.
